# Automatic interactive optimization for volumetric modulated arc therapy planning

**DOI:** 10.1186/s13014-015-0388-6

**Published:** 2015-04-01

**Authors:** Jim P Tol, Max Dahele, Jarkko Peltola, Janne Nord, Ben J Slotman, Wilko FAR Verbakel

**Affiliations:** Department of Radiotherapy, VU University Medical Center, De Boelelaan 1117, 1081 HV Amsterdam, The Netherlands; Varian Medical Systems, Paciuksenkatu 21, 00270 Helsinki, Finland

**Keywords:** Automatic treatment planning, Volumetric modulated arc therapy, OAR sparing

## Abstract

**Background:**

Intensity modulated radiotherapy treatment planning for sites with many different organs-at-risk (OAR) is complex and labor-intensive, making it hard to obtain consistent plan quality. With the aim of addressing this, we developed a program (automatic interactive optimizer, AIO) designed to automate the manual interactive process for the Eclipse treatment planning system. We describe AIO and present initial evaluation data.

**Methods:**

Our current institutional volumetric modulated arc therapy (RapidArc) planning approach for head and neck tumors places 3-4 adjustable OAR optimization objectives along the dose-volume histogram (DVH) curve that is displayed in the optimization window. AIO scans this window and uses color-coding to differentiate between the DVH-lines, allowing it to automatically adjust the location of the optimization objectives frequently and in a more consistent fashion. We compared RapidArc AIO plans (using 9 optimization objectives per OAR) with the clinical plans of 10 patients, and evaluated optimal AIO settings. AIO consistency was tested by replanning a single patient 5 times.

**Results:**

Average V95&V107 of the boost planning target volume (PTV) and V95 of the elective PTV differed by ≤0.5%, while average elective PTV V107 improved by 1.5%. Averaged over all patients, AIO reduced mean doses to individual salivary structures by 0.9-1.6Gy and provided mean dose reductions of 5.6Gy and 3.9Gy to the composite swallowing structures and oral cavity, respectively. Re-running AIO five times, resulted in the aforementioned parameters differing by less than 3%.

**Conclusions:**

Using the same planning strategy as manually optimized head and neck plans, AIO can automate the interactive Eclipse treatment planning process and deliver dosimetric improvements over existing clinical plans.

## Background

An important goal in radiotherapy treatment planning is relatively homogenous irradiation of the planning target volumes (PTVs) whilst minimizing dose to nearby organs-at-risk (OARs). In many situations, especially when there is a complex OAR - PTV geometry, this can be achieved using some form of intensity modulated radiation therapy (IMRT), including volumetric modulated arc therapy (VMAT). Planning IMRT and VMAT treatments requires optimization of multileaf collimator (MLC) leaf positions to achieve suitable dose distributions. Different treatment planning systems (TPS’s) offer different algorithms and interfaces to perform this optimization. For instance, some TPS’s allow for interactive optimization, which involves presenting dose-volume histograms (DVHs) to the user and dynamically updating them while the user adapts specific dose-volume objectives during optimization. Ultimately, many factors influence the final treatment plan, including acceptance criteria for PTV dose coverage and homogeneity, optimization objectives and weightings for OARs and PTVs. If the specific TPS allows for interactive optimization, the experience of the planner, along with the interaction between the planner and the TPS, can also influence the obtained plan quality and may contribute to large variations between planners and centers [1,2].

Treatment planning has become increasingly complex over the years, particularly regarding the number of OARs that are included in the optimization. For example, radiotherapy treatments for head and neck cancer evolved from essentially contralateral parotid gland and spinal cord sparing [3] to include sparing of the ipsilateral parotid gland, the contralateral submandibular gland, multiple swallowing muscles and the oral cavity [4]. This further increases the difficulty of plan optimization and increases the likelihood that inconsistent planning results are obtained between planners. Automated planning techniques might assist in reducing such variation and allow for the creation of more consistent and high quality plans. Although automated planning is in its infancy, promising results have already been obtained using knowledge-based planning [5-13] and automated multicriteria plan optimization [14-16].

One of the most commonly used TPS’s is Eclipse™ (Varian Medical Systems, Palo Alto, USA) which allows for interactive optimization of IMRT and VMAT plans. In our experience, when trying to create a plan that provides maximum OAR sparing for an individual patient (as opposed to creating plans where optimization stops once a pre-determined level of OAR sparing has been achieved, regardless of whether or not it could be improved upon for a given patient), the lowest achievable OAR doses and the trade-off between OAR sparing and PTV dose homogeneity [17] are typically not known in advance of making the plan. This means that the optimal settings of the optimization objectives have to be determined during planning. This can be done during interactive optimization. As a result, interaction between the planner and the TPS is a key step in producing a good plan, while at the same time it presents a source of considerable variation in the manual planning process. We propose a novel approach to automate interactive VMAT planning using the Eclipse TPS that aims to address the increasing challenges of manual planning and attempts to meet the competing demands for consistent and high quality planning under conditions of increasing complexity, while limiting the total planning time. We believe that these challenges are common to many centers, especially where the creation of complex treatment plans is concerned. This report describes our automatic interactive optimizer solution (AIO) and presents an initial evaluation of its performance.

## Methods

AIO was evaluated by creating simultaneous integrated boost plans for ten head and neck cancer patients that were previously treated using RapidArc®. RapidArc is the VMAT approach of Varian Medical Systems, based on the work of Otto [18]. Prescribed doses were 54.25Gy to the elective PTV (PTV_E_) and 70Gy to the boost PTV (PTV_B_) in 35 fractions of 1.55Gy and 2Gy, respectively. A 5mm transition zone (PTV_T_) was created between PTV_B_ and PTV_E_ to facilitate a dose fall-off between them. Table 1 shows the tumor site, stage and PTV volumes for each patient. For optimization and reporting purposes, a 6mm ‘virtual’ bolus region was used to obtain adequate target coverage in areas where the PTV approached the surface [19].

**Table 1:** **Detailed information of the included head and neck cancer patients**

**Patient number**	**Disease site**	**Stage**	**PTV**_**E**_ **(cm** ^**3**^ **)**	**PTV**_**T**_ **(cm** ^**3**^ **)**	**PTV**_**B**_ **(cm** ^**3**^ **)**	**Comp**_sal_^*^ **(cm** ^**3**^ **)**	**Comp**_**swal**_^**†**^ **(cm** ^**3**^ **)**	**Oral cavity (cm** ^**3**^ **)**
1	Larynx	T2N2c	428.7	93.7	243.3	66.7	7.1	-
2	Oropharynx	T2N2b	441.7	48.3	188.6	82.3	16.5	-
3	Oropharynx	T2N2a	240.6	43.4	94.5	42.4	25.0	-
4	Oropharynx	T4N1	288.2	57.3	237.5	60.6	10.6	-
5	Oropharynx	T4aN1	280.8	79.5	164.5	78.9	32.0	36.7
6	Oropharynx	T4aN2b	288.2	57.3	237.5	60.6	10.6	14.6
7	Oropharynx	T4aN1	360.4	62.9	143.7	73.0	35.0	57.1
8	Oropharynx	T3N1	500.3	87.9	231.7	55.0	11.9	29.4
9	Oropharynx	T4aN2c	258.4	155.7	328.9	38.3	4.8	38.1
10	Hypopharynx	T2N3	264.0	137.0	607.0	70.8	10.3	60.6

The disease site, stage and volumes of the elective, transition and boost planning target volumes (PTV_E_, PTV_T_, PTV_B_ respectively) and the volumes of the composite salivary and swallowing structures for all patients. The oral cavity was included as an organ-at-risk in 6/10 patients.

**Abbreviations:**

^*^Comp_sal_ = Composite salivary glands. Depending on degree of overlap with the PTVs and choice for inclusion by the treating clinician, comp_sal_ could consist of some, or all, of the ipsilateral and contralateral parotid and submandibular glands.

^†^Comp_swal_ = Composite swallowing muscles. Could consistent of some, or all, of the upper esophageal sphincter, upper and lower parts of the larynx, the superior, medial and inferior pharyngeal constrictor muscle, the cricopharyngeal muscle and the esophagus.

In all plans included in this study, optimization was performed using the progressive resolution optimizer (PRO) version 10.0.28 and followed by a ‘continue previous optimization’ (CPO) to improve PTV dose homogeneity [17]. Dose calculation was performed using the anisotropic analytical algorithm (AAA) version 10.0.28 with a 2.5mm grid size.

### The Progressive Resolution Optimizer (PRO)

PRO is used to optimize the MLC apertures of arc fields in a treatment plan. A multi-resolution (MR) model is used, meaning that the angular dose representation starts with a crude approximation that gets finer as the optimization progresses [20]. Input parameters are geometric characteristics of each field and a set of optimization objectives, see below, that can be adapted at any point during the optimization. The output of the optimizer is a control-point (cp) sequence, defining MLC configuration and MU count at each of the arc’s 178 control points. Each structure’s DVH-line is displayed and can be manipulated by adapting the optimization objectives, to attempt to meet clinical goals for PTV dose coverage and OAR doses. Each optimization objective has four input parameters: an optimization priority (P), a 2D-position on the DVH-graph representing the dose and volume goal (dose_goal_, volume_goal_), and information describing whether the dose_goal_ is an upper (maximum) or lower (minimum) dose limit for the structure’s DVH-curve.

The objective weighting (objective_weight_) is derived from P using a heuristic power law-formula. A doubling of P results in a 32 time multiplication of weight_objective_. To reduce the occurrence of hot and cold spots, PRO also increases objective_weight_ for objectives with volume_goal_ values of 0% or 100%.

Each point (i) inside a structure, not fulfilling the objective, gets assigned a penalty cost. A structure with n points and m optimization objectives obtains a total cost value of:

1 $$ \frac{{\displaystyle {\sum}_{j=1}^m}{\displaystyle {\sum}_{i=1}^n} objectiv{e}_{weight,j}\bullet {\left(dos{e}_i-dos{e}_{goal,j}\right)}^2}{n} $$

**Figure 1:**
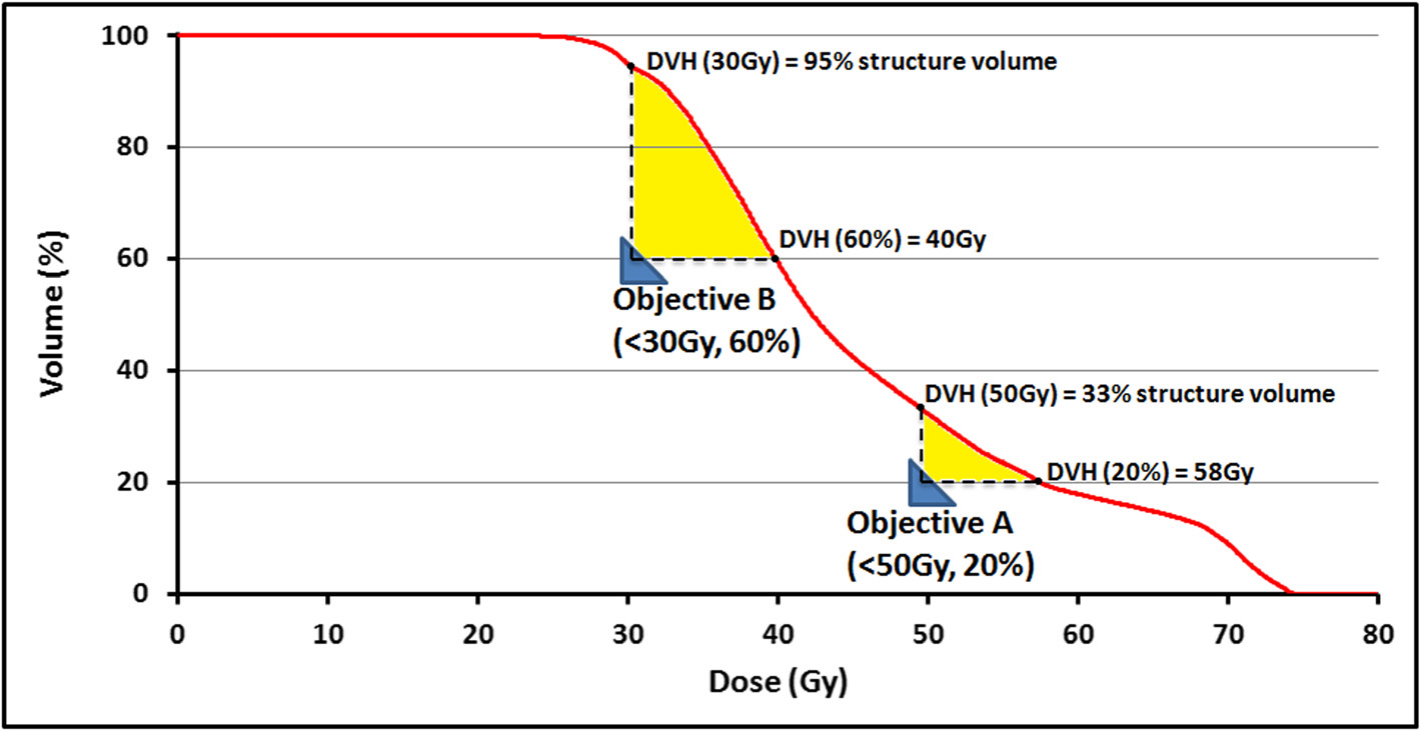
**Local force exerted on the DVH-curve by two optimization objectives.** A schematic representation of two optimization objectives (blue triangles) exerting force on the DVH-line (yellow area). Because structure points only contribute to an objective’s cost function if the set dose-volume criteria are violated, the objectives can lower the DVH-curve locally. Objective A contributes 13% (33%-20%) of structure points to calculating the cost function, with dose_i_ values ranging from 50-58Gy. Because objective B is placed further from the DVH-curve, the number of included structure points is larger (35%), with doses ranging from 30-40Gy. In case of overlap between the yellow areas, the included structure points would contribute to two separate cost functions.

The cost for an objective j is only taken into account for the range of voxels that violate the assigned dose-volume criteria. For example, if dose_goal_ and volume_goal_ are set to <5Gy and 10%, respectively, 10% of the structure volume may have doses higher than 5Gy without contributing to the cost function for j. If this criterion is violated, the cost function is calculated for the structure points with dose_i_ values ranging from 5Gy to the dose achieved at 10% volume of the DVH-curve, see Figure 1. The total cost function value is calculated by summing equation (1) over all structures that are included in the PRO optimization.

During optimization, new, mutated MLC configurations that result in the largest decrease to the total cost function are successively added to the control points. In the first MR-level, the optimizer divides the arc into eleven 16-cp long sequences. The optimizer randomly selects 8 sequences and makes simultaneous changes to MLC configurations, while taking into account physical limitations such as MLC leaf speed, gantry speed and dose rate. Three subsequent increases in the MR-level increase the number and while decreasing the size of these cp-sequences to 22 times 8-cps, 44 times 4-cps and finally 88 times 2 cps. Changes in positioning of the MLC leafs are dictated by set of 0-8 cp-sequences found to contribute most to reducing the total cost function. These changes are incorporated into the subsequent iterations. At the start of each iteration, the optimization objectives, which might have been changed, are sent to the PRO algorithm, allowing it to find more optimal MLC configurations to satisfy the set objectives. If the total cost function has converged, the optimization automatically proceeds to the next MR-level. The user can pause the automatic progression to subsequent MR-levels allowing the user more time to make changes to the dose-volume objectives. In the earlier MR levels, PRO is more flexible towards changes in the optimization goal during optimization, as it can make large changes in the MLC leaf configurations at each iteration. The possibility to adjust to changed dose-volume objectives gets smaller in later MR levels as the modified cp-sequence length decreases.

### Optimization using dose-volume objectives

Due to the power-law nature of the objective weightings, structures with higher prioritized objectives will have substantially more influence on the resulting plan. In our clinical planning protocol, described below, optimization objectives of PTV structures are assigned higher priorities values than OAR objectives. OAR objectives can therefore be gradually pushed to lower dose values at each iteration during optimization without significantly affecting the DVH’s of the PTVs. This concept forms the basis of our institutional approach to manual interactive optimization and our automation of it, explained below.

### Interactive optimization

**Figure 2:**
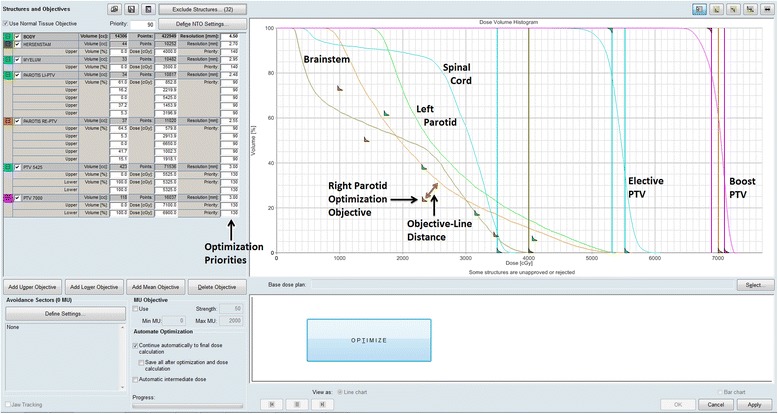
**Eclipse optimization window for a simple head and neck cancer patient.** Eclipse optimization window for a simple head and neck cancer patient showing DVH-lines and optimization objectives of the elective and boost PTVs, spinal cord and brainstem, and two OARs (left and right parotids).

PRO uses a simplified fast dose calculation algorithm to display DVH lines during the optimization process (Figure 2), visualizing changes in PTV and OAR doses while optimization of the MLC leaf positions is being performed. As previously explained, the priority and distance (i.e. dose difference) of an optimization objective to its corresponding DVH-line determines the ‘effort’ made by the optimization algorithm to meet this objective. Initial evaluation of the algorithm during the clinical introduction of RapidArc at our department in 2008 resulted in the current institutional planning approach for head and neck tumors [21-23]. Because the patient-specific favorable dose-volume values of the OAR optimization objectives are typically not known at start of planning, this approach involves continuous user interaction, adapting the location of the OAR objectives so that they maintain a certain diagonal distance to their respective DVH-line while the optimization progresses through the different MR-levels. Parallel OAR, such as the parotid glands, submandibular glands and swallowing muscles, typically have 4-5 objectives, distributed along the volume axis of the DVH (prioritized at 80-90, but can be lowered for individual OAR after discussion between clinician and planner) of which 3-4 are interactively adjusted. Serial OAR such as the spinal cord and brainstem, have a single maximum point dose objective that remains unchanged during optimization. Objectives for the PTVs have priority values of 130 and remain unchanged during planning. Interactively adapting multiple objectives along a DVH results in equal effort/attention directed at sparing over the entire volume range and works well for parallel OAR in which the mean dose has been demonstrated to correlate with toxicity [24,25]. Since continuous pulling of the OAR objectives could lead to local underdosage of the PTVs near those OARs, parts of the PTV within 5mm of the OARs are defined as an extra PTV volume with separate minimum dose objectives. Interactive optimization using this approach creates treatment plans with a high probability of satisfying our institutional guidelines for coverage and homogeneity of the PTV. The aims are that at least 99% of the boost volume is covered with 95% of the prescribed dose (V95 ≥ 99%) and no more than 4% should receive a dose greater than 107% (V107 ≤ 4%). Corresponding aims for the elective volume are V95 ≥ 98% and V107 ≤ 15%.

Head and neck cancer treatment plans currently include 2 PTVs planned to receive different doses, and up to 12 individual salivary and swallowing structures are routinely used. In addition, the oral cavity was included during optimization for 6/10 patients. Because so many OARs are included, the continuous manual adjustment of multiple optimization objective locations along numerous DVH lines has become increasingly challenging.

### Automatic optimization solution

**Figure 3:**
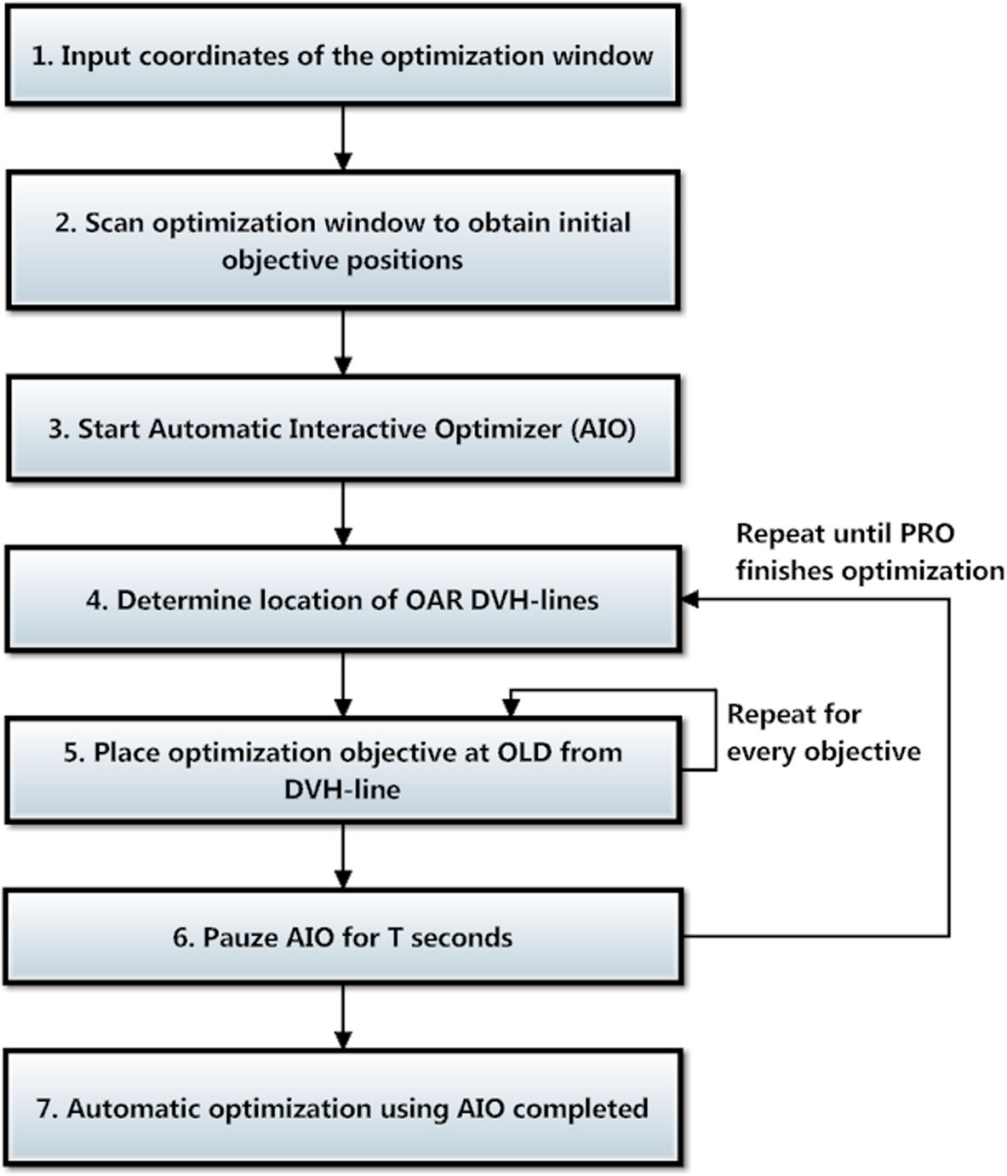
**Flowchart of the automatic interactive optimizer (AIO).**

An automated alternative to manual interaction was developed to interactively optimize a large number of OARs consistently (automatic interactive optimizer, AIO). AIO was coded in Lazarus, a free Pascal compiler (http://www.lazarus.freepascal.org/). The flowchart in Figure 3 provides an overview of an automated optimization using AIO. Because Eclipse does not allow for modifications of the optimization algorithm and interface, AIO was designed to take over interactivity of the user by automatically moving the cursor and adapting the position of the OAR objectives on the screen. If Eclipse would permit access to the optimization algorithm, this process could easily be implemented in the optimizer itself.

**Figure 4:**
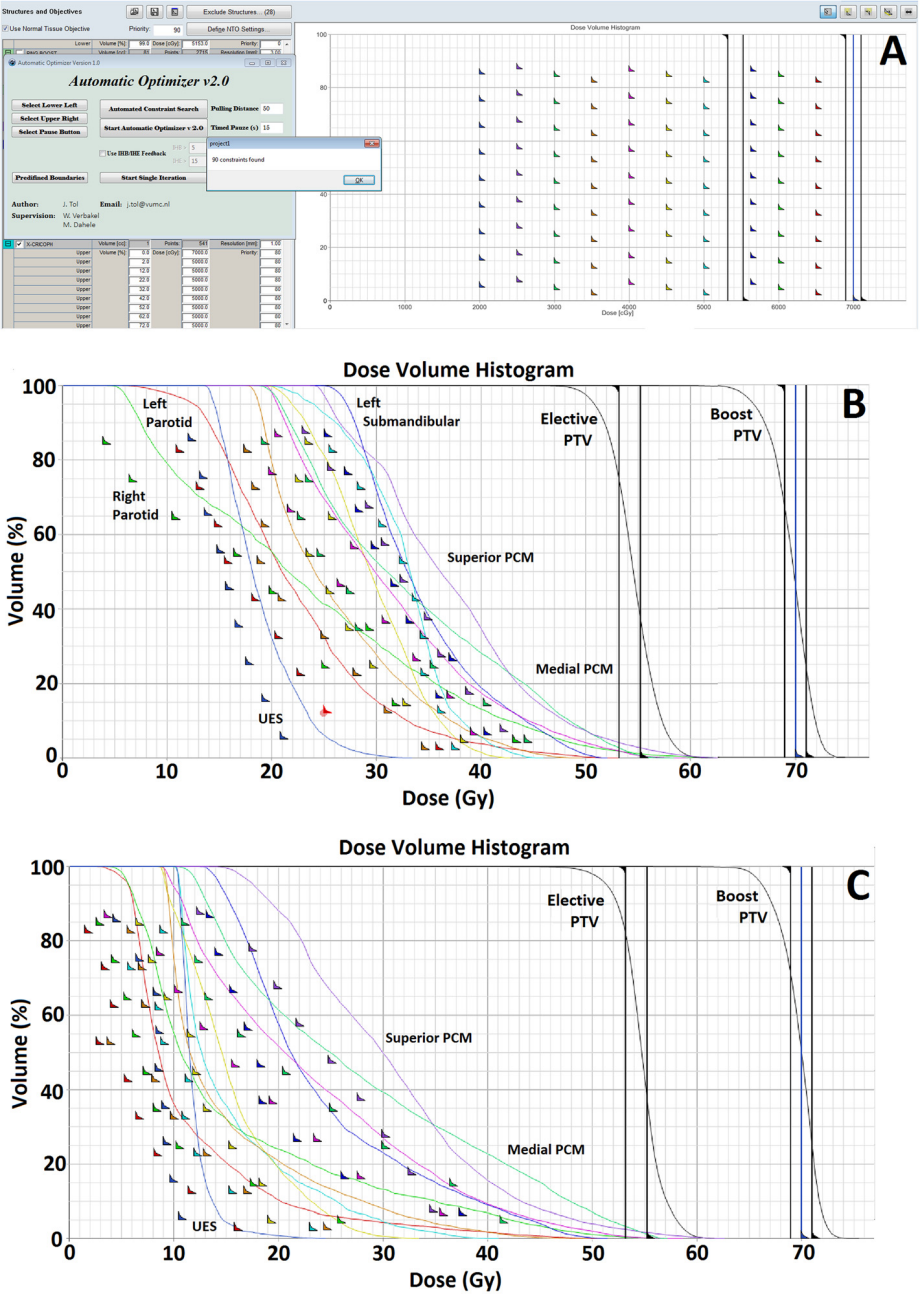
**Different stages of optimization using the automatic interactive optimizer (AIO). A)** shows the initial position of all OAR optimization objectives along with the graphical user interface of the AIO. **B)** shows the optimization window after 3 minutes while the AIO is being performed on a complex head and neck case. **C)** shows the optimization window after 11 minutes. In this case, the full optimization took approximately 29 minutes. Shown abbreviations: PTV = Planning target volume, PCM = Pharyngeal constrictor muscle and UES = Upper esophageal sphincter.

Before the PRO optimization is started, the user inputs the coordinates of the optimization window (step 1, Figure 3), AIO scans this window determining the initial location of all OAR objectives (step 2, Figures 3 and 4A). Because color-coding is used to differentiate between the various OAR objectives, each OAR should be assigned a unique color. When the optimization is started (step 3, Figure 3), AIO scans the optimization window and determines the location of every DVH-line (step 4, Figure 3), based on the unique color of each OAR. AIO then places every OAR objective at a fixed diagonal pixel distance (the so-called ‘objective-line’ distance [OLD]) from the respective DVH-line (step 5, Figures 3 and 4B&C). Because AIO moves the optimization objectives as soon as the DVH-lines appear on the screen, their initial locations do not influence the resulting plan quality. If the new objective position is already occupied with a different optimization objective, AIO searches for the next available position while keeping a minimum distance of 20 pixels between the objectives to avoid overlap. To allow convergence of the cost function and let PRO advance to the next MR-levels in the optimization process, AIO is paused for T seconds (step 6, Figure 3), after which steps 3-5 are repeated until PRO completes the RapidArc optimization (step 7, Figure 3).

By automatically determining the position of the OAR DVH-lines and repositioning the optimization objectives, AIO removes the limitation that is posed by the relative knowledge/experience, precision and attention of the planner in the interactive planning process, allowing far more frequent and consistent updating of the OLD. If the OLD value is equal for each OAR, consistent positioning of the optimization objectives using AIO results in a constant cost function for each OAR objective throughout the optimization process. This allows PRO to balance sparing of the various OARs, depending on the assigned optimization priorities. The OLD and PTV/OAR optimization priorities can be adjusted in order to meet user-specific criteria for PTV dose coverage and homogeneity [26]. Additionally, because the location of every OAR objective is adjusted automatically, more optimization objectives can be used simultaneously throughout the optimization. Since the dose_goal_ and volume_goal_ values of these objectives determine the range of dose values taken into account when calculating the cost function (see Figure 1), distributing optimization objectives over the entire volume range may offer improved sparing compared to using 3-4 objectives per OAR.

### Evaluation endpoints

For each patient, the manually optimized (clinical) plan is compared to the (nominal) AIO plan created using 9 different optimization objectives per OAR, an OLD value of 50 pixels and a pause time T of 15 seconds. The OLD value is screen-dependent, a value of 50 was chosen to reflect the OLD used clinically and corresponds to roughly 5% of the width of the optimization window at a screen resolution of 1980x1080 pixels. In the AIO plans, the optimization priorities of PTV and OAR objectives are kept equal to those used in the clinical plans. To investigate consistency of AIO plans, a single patient was re-planned 5 times using the same AIO settings. To determine the influence of the initial placement of the optimization objectives on the resulting plan, optimization of 5 patients was performed again without interactively repositioning the optimization objectives. The resulting optimization objectives in the nominal AIO plans were used as the initial location of the optimization objectives in these non-interactively optimized plans, and remained unchanged throughout the optimization.

To evaluate resulting plan quality when using different settings of the AIO, additional plans were created using (i) 3 and 6 planning objectives per OAR, (ii) OLD values of 25 and 75 pixels and (iii) pause times of 7.5 and 30 seconds while the other parameters were kept at the aforementioned values.

The following dosimetric parameters were evaluated after normalizing the AIO plans to deliver the same mean dose to PTV_B_ as the respective clinical plan: 1) Mean doses to the oral cavity (D_OC_), individual and composite salivary glands (D_sal_) and swallowing muscles (D_swal_), 2) PTV_B_ and PTV_E_ volumes receiving less than 95% (V95) and more than 107% (V107) of the prescribed dose. The size of the oral cavity, composite salivary (comp_sal_) and swallowing structures (comp_swal_) is shown in Table 1. To determine whether improved sparing using AIO resulted in increased dose deposition in the remainder of the body, the mean dose to a body-PTV structure (obtained by subtracting the combined PTV from the body contour) was determined, along with the body-PTV volume receiving >5Gy (V5Gy), >30Gy (V30Gy) and >50Gy (V50Gy).

## Results

AIO and clinical plan results were averaged over all 10 patients and summarized in Table 2. Maximum spinal cord and brainstem doses were similar and considered clinically acceptable in all plans. Although PTV_B_ and PTV_E_ V95 values were marginally worse, consistently improved OAR sparing was obtained in the nominal AIO plan that used an OLD of 50 pixels, 9 optimization objectives per OAR, and a pause time T of 15 seconds. Compared to the clinical plan, the nominal AIO plan reduced mean dose to individual salivary structures by on average 0.9-1.6Gy, and reduced D_sal_ from 26.2 ± 5.5Gy to 25.0 ± 5.8Gy. The oral cavity and individual swallowing muscles saw a larger dose reduction, with composite metrics D_OC_/D_swal_ decreasing by 3.9Gy/5.6Gy, on average. This increased OAR sparing using AIO came at the expensive of a 0.1% higher body-PTV mean dose on average, while V5Gy, V30Gy and V50Gy were respectively 0.2%, 0.4% and 0.4% higher. AIO plans required 2.8% more monitor units (MU), going from an average of 492MU per 2Gy fraction in the 10 clinical plans to 506, suggesting that additional MLC modulation was needed to achieve improved OAR sparing.

**Table 2:** **Automatic Interactive Optimizer (AIO) and clinical plan results**

	**Plan**	**Clinical**	**Nominal AIO**	**AIO OLD = 25** ^**††**^	**AIO OLD = 75** ^**††**^	**AIO 3 objectives/OAR** ^**||**^	**AIO 6 objectives/OAR** ^**||**^	**AIO T = 7.5s** ^**¶**^	**AIO T = 30s** ^**¶**^
	**PTVB V95** ^*^ **(%)**	99.1 ± 0.2	98.9 ± 0.5	99.5 ± 0.2	97.5 ± 1.2	99.2 ± 0.5	98.9 ± 0.5	98.7 ± 0.6	98.5 ± 0.9
	**PTVB V107** ^*^ **(%)**	1.1 ± 1.4	1.2 ± 1.6	0.5 ± 0.6	3.4 ± 3.6	0.5 ± 0.7	1.1 ± 1.2	1.9 ± 1.9	1.6 ± 1.6
	**PTVE V95** ^*^ **(%)**	98.2 ± 1.1	97.7 ± 0.7	98.7 ± 0.6	96.0 ± 1.6	98.3 ± 1.0	97.8 ± 1.0	97.4 ± 1.0	97.3 ± 1.0
	**PTVE V107** ^*^ **(%)**	14.5 ± 3.8	13.0 ± 5.4	9.5 ± 4.6	16.5 ± 6.2	10.3 ± 4.7	12.2 ± 5.0	13.7 ± 5.6	13.6 ± 5.1
**Max dose (Gy)**	**Spinal Cord**	39.0 ± 3.7	39.1 ± 4.1	38.3 ± 4.0	39.8 ± 3.7	38.5 ± 4.2	38.8 ± 4.0	39.0 ± 3.8	38.3 ± 4.9
	**Brainstem**	37.7 ± 7.7	37.3 ± 8.0	37.7 ± 8.3	38.8 ± 7.8	37.0 ± 7.2	37.6 ± 8.3	37.0 ± 7.9	37.5 ± 8.3
**Mean dose (Gy)**	**Contralateral Parotid**	19.6 ± 5.6	18.7 ± 5.7	21.4 ± 6.9	17.6 ± 5.3	21.4 ± 6.7	19.5 ± 6.0	18.5 ± 5.5	19.0 ± 5.9
	**Ipisilateral Parotid**	31.2 ± 8.1	29.9 ± 8.4	32.0 ± 8.5	27.5 ± 7.1	32.1 ± 8.7	30.5 ± 8.3	29.5 ± 7.9	29.8 ± 8.1
	**Contralateral Submandibular**	31.2 ± 7.4	29.6 ± 8.0	34.0 ± 8.6	27.7 ± 7.3	33.9 ± 8.6	30.6 ± 8.3	29.4 ± 7.0	30.5 ± 7.4
	**Oral Cavity**	28.6 ± 7.0	24.7 ± 7.9	26.7 ± 8.7	23.6 ± 7.6	27.0 ± 10.3	25.6 ± 8.3	24.2 ± 6.9	26.1 ± 7.4
	**Comp**_**sal**_^**†**^	26.2 ± 5.5	25.0 ± 5.8	27.7 ± 6.5	24.2 ± 5.3	27.8 ± 6.2	25.8 ± 5.9	24.7 ± 5.4	25.2 ± 5.7
	**Comp**_**swal**_^**†**^	28.7 ± 8.0	23.1 ± 8.7	27.0 ± 9.5	21.7 ± 8.7	26.7 ± 9.5	23.8 ± 9.2	22.5 ± 8.8	23.4 ± 8.7

AIO and clinical plan results averaged over all 10 patients using different AIO settings. Clinically acceptable doses to the brainstem and spinal cord were achieved in all plans.

**Abbreviations:**

^*^PTV_B_, PTV_E_, PTV_T_: Boost, elective, transition planning target volumes, respectively.

^**†**^Comp_sal_ / Comp_swal_: Composite salivary glands / Composite swallowing muscles.

^**††**^AIO OLD = 25/75: AIO plan using an objective-line distance (OLD) of 25/75 pixels, a pause time of 15 seconds and 9 objectives per OAR.

^**||**^AIO 3/6 objectives per OAR: AIO plan using 3/6 objectives per OAR, a pause time of 15 seconds and an OLD of 50 pixels.

^**¶**^AIO T = 7.5/30s: AIO plan using pause times (T) of 7.5/30 seconds, 9 objectives per OAR and an OLD of 50 pixels.

**Figure 5:**
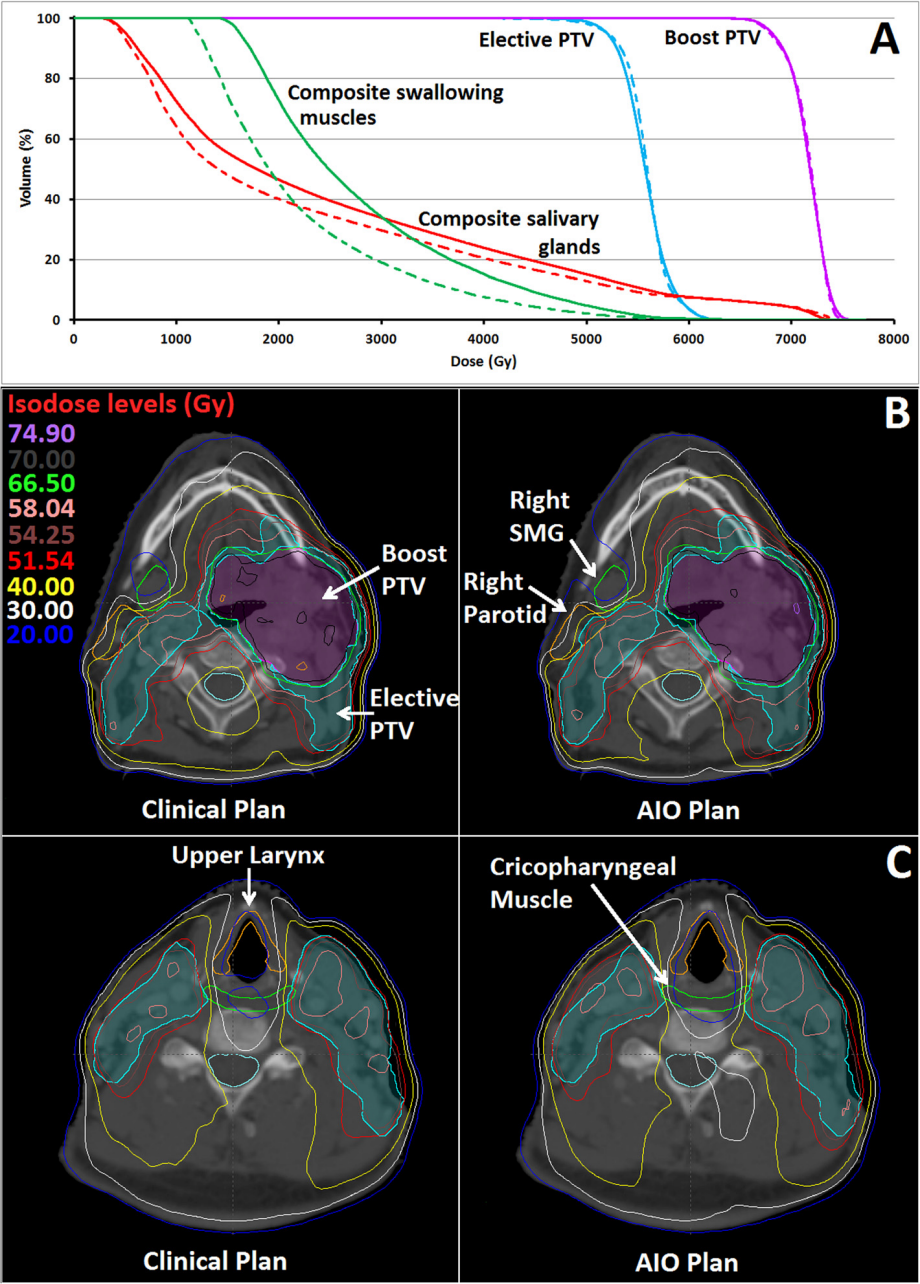
**DVH-lines and dose distributions of an automatic interactive optimizer (AIO) plan and clinical plan. A)** shows the DVH-lines of an AIO (dashed lines) and clinical (solid lines) plan for patient 2. Mean dose to the composite salivary glands (red) and swallowing muscles (green) decreased while approximately equal coverage of the boost and elective PTVs (magenta and cyan, respectively) was obtained. **B)** shows the dose distributions on the level of the salivary glands while **C)** shows the dose distributions on the level of the swallowing muscles.

Figure 5 shows typically resulting DVH-lines and dose distributions of a nominal AIO plan (triangles) and clinical plan (rectangles) for patient 2. Using AIO, D_sal_ and D_swal_ decreased by 2.3 and 5.7Gy, respectively while approximately equal coverage of the boost and elective PTVs was obtained.

Running AIO five times on a single patient showed variations in D_sal_ and D_swal_ no greater than 0.9% and 2.0%, respectively. V95 of PTV_B_ and PTV_E_ ranged from 98.6-99.0% and 97.1-97.7% respectively, while V107 ranged from 0.5-1.2% and 14.3-17.3%. For this patient, the PTV coverage values were slightly lower than the institutional guidelines because the AIO plans were normalized to receive the same mean PTV_B_ dose as the original clinical plan. In clinical practice this would have been solved by either adjusting the normalization by a fraction of a percent, or by running an additional CPO calculation while slightly increasing the priority on PTV optimization objectives. The non-interactive optimization of the nominal AIO plans of 5 patients resulted in PTV_B_/PTV_E_ V95% values within 0.6%/0.3% of their respective nominal AIO plan, while D_OC_/D_sal_/D_swal_ varied no more than 0.3/0.4/0.8Gy.

Using 3 instead of 9 optimization objectives per OAR was associated with an increase in D_OC_, D_sal_ and D_swal_ by 2.3Gy, 2.8Gy and 3.6Gy compared to the nominal AIO plan, respectively (Table 2), while PTV dose homogeneity improved. Running AIO with 6 objectives per OAR resulted in similar PTV coverage and homogeneity as the nominal plan, while D_OC_, D_sal_ and D_swal_ were respectively 0.9Gy, 0.8Gy and 0.7Gy higher. Although an OLD of 25 pixels improved V95/V107 of PTV_B_ and PTV_E_ by 0.6/0.7% and 1.0/3.5%, respectively, D_OC_/D_sal_/D_swal_ values increased by respectively 2.0/2.7/3.9Gy. The opposite was noticed using an OLD of 75 pixels, which degraded V95/V107 of PTV_B_ and PTV_E_ by 1.4%/2.2% and 1.7%/3.5%, respectively, while D_OC_/D_sal_/D_swal_ decreased by 1.1/0.8/1.4Gy.

Changing pause times of the AIO had little impact on the PTV coverage/homogeneity and OAR sparing. On average, AIO plans with a T = 7.5s achieved slightly lower OAR doses relative to T = 15s, while T = 30s increased them. This increase was associated with a decrease in average optimization time, which was approximately 56, 32 and 16 minutes for T values of 7.5, 15 and 30 seconds using an Intel® Xeon® E2520 2.40Ghz CPU in combination with a distributed calculation grid. Although the magnitude was influenced by the complexity of the patient, the number AIO iterations (steps 4 to 6, Figure 3) decreased for increasing T values. For patient 1 for example, AIO performed 85, 50 and 15 iterations using pause times of 7.5, 15 and 30 seconds, respectively.

## Discussion

This study presented an initial evaluation of our in-house developed automatic interactive optimizer (AIO). AIO was designed to automate our interactive optimization protocol for head and neck cancer using the Eclipse treatment planning system in order to improve plan quality and consistency. Under the planning constraints posed during optimization by the minimum/maximum dose objectives on the PTVs and single point dose objectives on serial OARs (e.g. spinal cord and brainstem), AIO gradually positions the optimization objectives of parallel OARs (e.g. salivary glands, swallowing muscles and oral cavity) at lower dose values throughout the optimization process. Compared to the clinical plans, nominal AIO plans achieved substantially improved sparing of the swallowing muscles and oral cavity, although a small decrease in PTV dose homogeneity was found in some patients, which was considered clinically acceptable [17,26]. On the other hand, if it is desired, then with minimal loss in OAR sparing, the PTV homogeneity can be improved after the AIO optimization by running a relatively short (non-interactive) CPO with higher priorities on the PTV optimization objectives. It is important to state that the use of AIO does not result in any changes in the quality assurance process. Even though AIO resulted in dosimetric improvements over clinical plans, the final DVHs and dose distributions should still be routinely evaluated by the planning dosimetrist, radiation oncologist and physicist, prior to acceptance for treatment. This is no different from treatment plans created manually or using different automatic planning approaches.

Once AIO is running, it is fully automated, thereby allowing the planner to invest time in other activities while the optimization is being performed. Running AIO optimizations in a virtual machine (Oracle VirtualBox; http://www.virtualbox.org) allows the user to retain control of their cursor. They can therefore continue using their PC while AIO is running. A virtual machine also allows a custom screen resolution to be defined. Larger resolutions may prevent potential overlap of the optimization objectives during optimization, which leads to suboptimal placement.

The effect of changing several user-definable AIO parameters was investigated and showed: 1) The selected objective-line distance (OLD) influences the trade-off between PTV dose homogeneity and OAR sparing. These results can be expected because in PRO, increasing or decreasing the OLD will relatively increase or decrease the cost of these OAR optimization objectives, compared to the PTV objectives. 2) An increased number of optimization objectives generally improved OAR sparing because a reduction in OAR doses was attempted at a greater range of structure volumes. 3) The desired balance between optimization time and resulting plan quality is determined by the chosen value of T. Larger values for T resulted in less AIO iterations and in effect, the optimization objectives were not placed at a constant distance from their DVH-line at all times. Lower values of T increase the number of AIO iterations and optimization time, allowing the optimizer in theory to minimize the cost function better. However, no marked improvement was noted using T = 7.5s instead of 15s in this study.

Nominal AIO plans, generated with an OLD of 50 pixels, 9 optimization objectives per OAR and a T value of 15 seconds, provided a good balance between optimization time, OAR sparing and PTV dose homogeneity, and would therefore provide a reasonable starting point for treatment planning. The present results can also be generalized to manual interactive planning in the Eclipse TPS. The initial ‘grid’ of optimization objectives that was used (Figure 4A) for example, indicates that AIO does not need a proper initial prediction of the objective starting locations to obtain high quality plans, as long as the optimization objectives are repositioned when the DVH-lines appear in the optimization window. This assumption was validated by re-optimizing the nominal AIO plans of 5 patients non-interactively, using the final placement of the interactively determined optimization objectives. The resulting plans provided similar OAR sparing to the interactively optimized AIO plans.

AIO was designed to automate interactive optimization in the Eclipse TPS. We do not perceive this as a weakness, as many automated planning solutions will ultimately be vendor or TPS specific. However, since VMAT optimization algorithms generally start with coarse MLC configurations that get progressively finer as the optimization progresses in one way or another, the core concept of AIO, pushing OARs to lower doses throughout the optimization process while the MLC apertures are being defined, may also be transferable to different TPS’s. Although AIO was only used in combination with our clinical OAR and PTV optimization priorities so that it generated plans fulfilling our clinical plan acceptance criteria, we expect it to work equally well for other criteria by adjusting the optimization priorities for PTVs and the OLD. Running the same plan several times on the same patient, AIO demonstrated a satisfactory degree of consistency in OAR sparing and PTV dose homogeneity, demonstrating the reproducibility of the automated optimization approach [26]. Because of the consistent placement of the optimization objectives throughout the optimization, we expect that AIO optimized plans are well suited for treatment planning comparisons and planning studies where generally the user dependency of setting and interacting with parameters can substantially influence plan results [1].

Some potential limitations deserve comment. The present study focused on the evaluation of AIO for VMAT head and neck cancer planning. This was because VMAT is our standard delivery method for intensity modulation and head and neck is one of the sites where planners have to routinely deal with many different OARs during interactive optimization. We did not use the latest version of PRO. The version used in the study (v10.0.28) is the one that is clinical use in our department. Preliminary tests indicate that AIO can also be used with newer versions of PRO (11.0.31–13.5.10). The current analysis investigated replacing manual interaction with software-controlled interaction for a particular planning strategy in which constant OLD are used and priorities are held constant. This was done to replicate our current institutional planning strategy. This strategy also requires that the user knows the input priorities that generally result in acceptable plans for the majority of patients. We have used a standard set of priorities for PTV and OAR, derived from our clinical practice, and we have not attempted to investigate the influence of applying different priorities. Although AIO has been specifically designed to reduce mean doses of OARs, other planning strategies (e.g. focused on high or low dose reduction) can also be incorporated. AIO was designed to automate an important step in planning, it does not automate the complete planning process. Optimality of a plan may depend on more than the optimization alone: the number of (partial) arcs [27], chosen collimator angles, isocenter positions and jaw settings, all which have to be input by the user, may also influence the achieved plan dosimetry. In addition, we recognize that there may be selected cases where planning teams may wish to use manual interaction. The use of AIO does not prohibit switching to manual optimization if desired.

Another automated planning solution described in the literature is automated multi-criteria optimization, typically using a ‘wish list’ that contains PTV and OAR optimization priorities and weights. For every OAR, weightings are successively increased until either the required OAR sparing is achieved or the specified PTV dose homogeneity/sparing of a higher prioritized OAR is compromised [14-16]. The downside of this approach could be that it prevents reduction of dose to low prioritized OAR, even though a substantial reduction could be obtained with only a minor increase in dose to a higher prioritized OAR. This does not occur when using AIO because, apart from the optimization objectives that are assigned, all the optimization objectives are continuously pushed to lower doses. This is illustrated in a patient where AIO decreased the mean dose of three different swallowing muscles by 5.5Gy, while increasing mean dose to the contralateral submandibular gland by 1.8Gy. Although the clinician should decide whether this is trade-off is deemed acceptable, it illustrates that a small increase in dose to a single OAR may lead to a larger dose reduction in multiple OARs.

## Conclusions

The present report described the development and initial evaluation of AIO, automating and improving the interactive optimization process in the Eclipse TPS. AIO consistently allows the user to more fully exploit the potential of interactive optimization in Eclipse. Importantly, this intermediate interface requires no modification of the TPS. Preliminary testing shows that AIO can deliver dosimetric improvements over manually optimized head and neck plans. AIO has been clinically implemented for head and neck treatment planning.
